# An investigation of a measles outbreak in Japan and Taiwan, China, March–May 2018

**DOI:** 10.5365/wpsar.2018.9.2.005

**Published:** 2018-08-22

**Authors:** Kazuki Shimizu, Ryo Kinoshita, Keita Yoshii, Andrei R. Akhmetzhanov, Sungmok Jung, Hyojung Lee, Hiroshi Nishiura

**Affiliations:** a Graduate School of Medicine, Hokkaido University.

## Abstract

**Objective:**

To investigate a measles outbreak that spread to Japan and China, Taiwan, China during March–May 2018, exploring the characteristics of the super-spreading event.

**Methods:**

A contact investigation of the index case and reconstruction of the epidemiological dynamics of measles transmission were conducted. Employing a mathematical model, the effective reproduction number was estimated for each generation of cases.

**Results and discussion:**

A single index case gave rise to a total of 38 secondary cases, 33 in Japan and five in China, Taiwan, China. Subsequent chains of transmission were observed in highly vaccinated populations in both Japan and China, Taiwan, China. The effective reproduction number of the second generation was > 1 for both Japan and China, Taiwan, China. In Japan, the reproduction number was estimated to be < 1 during the third generation. Vaccination of susceptible individuals is essential to prevent secondary and tertiary transmission events.

## Introduction

Measles is caused by the measles virus, a single-stranded negative-sense enveloped RNA virus. It is a vaccine-preventable disease, subject to control and elimination via surveillance and vaccination programmes. (*1*, *2*) Since the year 2000, a two-dose schedule for measles vaccination has been recommended by Global Measles Mortality Reduction and Regional Elimination: Strategic Plan, (*2*) organized by the World Health Organization (WHO), United Nations Children’s Fund and the United States Centers for Disease Control and Prevention, and substantial progress has been made towards measles elimination in countries that belong to the WHO Western Pacific Region. (*3*, *4*) Nevertheless, the virus has continued to circulate, causing multiple outbreaks in member countries and surrounding areas. (*3*-*5*) Since 2017, there has been a surge of global measles cases, especially in European countries, (*6*) and the chance of experiencing an outbreak in the Western Pacific Region has continued to be to be high.

Even in highly vaccinated countries such as Japan, (*7*) imported cases can produce clusters with multiple chains of transmission. (*8*) Interrupting these chains requires supplementary vaccination among adults, especially those who are unvaccinated or have received only one vaccination. (*5*, *9*) If susceptible groups of people remain unvaccinated, countries are at risk of experiencing outbreaks with additional introductions of imported cases. On 23 March 2018, the Japanese Government was notified of an imported case of measles in Okinawa prefecture, the southernmost prefecture of Japan, arising from a China, Taiwan Chinese traveller. (*10*) An outbreak of measles occurred in Okinawa, arising from the contact with the index case; moreover, there have been chains of transmission arising from the same index case in China, Taiwan, China. (*11*)

The present study aims to investigate a cross-border outbreak of measles that spread to Japan and China, Taiwan, China and describe the dynamics of disease transmission in this outbreak.

## Methods

### Case definition and epidemiological data collection

A measles case was defined by the presence of (1) a generalized rash, (2) fever, and (3) other typical symptoms including cough, coryza and conjunctivitis and by laboratory confirmation of measles infection. Laboratory-confirmed measles is defined as the detection of measles-specific immunoglobulin M (IgM) antibodies in patient serum (*12*) or detection of virus by nested real-time polymerase chain reaction (PCR). Modified measles is defined by the presence of at least one of the three signs or symptoms described above plus laboratory confirmation of measles infection. In general, modified measles is a milder form of the disease with a longer incubation period (14–20 days) and a lack of premonitory symptoms, Koplik spots or a generalized rash. (*13*) The rash, when it occurs, can be localized to a foot or hand. Modified measles is less infectious than typical measles infection, but those with modified measles can spread the infection to others and still require watchful observation. (*14*)

The present study is based on governmental reports of the outbreak investigations in Japan and China, Taiwan, China. (*10*, *15*) We retrospectively scanned all real-time reports of the outbreak, including those from local prefectures that were affected: Okinawa, Aichi, Kanagawa and Tokyo in Japan. (*16*-*19*) We then reconstructed the transmission dynamics of the measles outbreak that arose from the index case. Dates of illness onset and laboratory confirmation, age, sex, country or area of residence and vaccination history of cases were retrieved, allowing us to characterize the epidemic as a function of these variables. The index case’s entry and exit dates, to and from Japan and China, Taiwan, China, and a detailed history of potential contacts were retrieved from publicly available information. (*15*, *16*) Using this information, we characterized the descriptive epidemiological features of the epidemic.

### Effective reproduction number

Effective reproduction number (*R_n_*), interpreted as the average number of secondary cases generated by a single primary case in generation *n*, is an objective epidemiological measure to quantify disease spread between generations. An *R_n_* value > 1 reflects an increase in the number of cases, while an *R_n_* value < 1 ensures that the number of cases is decreasing. The temporal distribution of *R_n_* values could thus reflect an outbreak brought under control; therefore, the measure can be used for assessing the effectiveness of public health interventions and/or for designing disease control policy in the future, such as restricting human movements during the course of an epidemic or raising awareness among the general public.

Because the mean generation time of measles infection is relatively long compared to its variance (mean: 11.7 days and variance: 9.0 days^2^), (*20*) we estimated a generation-specific *R*_n_ in the present study. We first reconstructed the transmission network using publicly available information. Subsequently, we calculated the generation-dependent number of cases by either referring to the contact history or imposing an assumption that the interval between generations was constant at 11 days. To assign cases into a particular generation, first we relied on the known contact history (the link to a primary case) that was available in the case reports. We confirmed that the peaks of the generation-specific epidemic curve were about 11 days apart from each other, and then we separated cases into different generations by referring to contact-tracing results. If the transmission tree was not known, we took the mid-point between peaks as the cut-off date separating two generations. As part of the sensitivity analysis, we also calculated the number of cases in each generation, including ± 1 day of the imposed cut-off date. Given the number of cases in the *n*th generation, *c_n_*, the expected number of cases in the (*n* + 1)th generation was modelled as:

**Figure:**
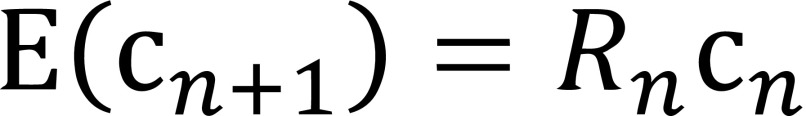

where *R*_n_ represents the effective reproduction number of the *n*th generation which represents the average number of secondary cases generated by a single primary case. We assumed that the observed number of generation-dependent cases followed a Poisson distribution:

**Figure:**
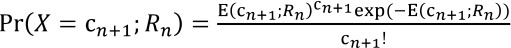


Maximum likelihood estimation was employed to appropriately quantify the uncertainty (confidence intervals) of parameters. Alternatively, we could have identified a point estimate of the *R*_n_ by counting the number of cases in each generation and taking the ratio of the number of cases in adjacent generations. Using the second equation as the likelihood function, the maximum likelihood estimate of parameters was calculated to obtain parameter estimates of the *R*_n_, and the 95% confidence interval (CI) was computed by using the profile likelihood method.

### Data availability

In addition to provided data sources, (*15*-*19*) the collected information of cases is shared on an open online repository. (*21*)

### Ethics

This study was based on publicly disclosed information as part of an outbreak investigation and did not require ethical review.

## Results

### Index case and contacts

**Fig. 1:**
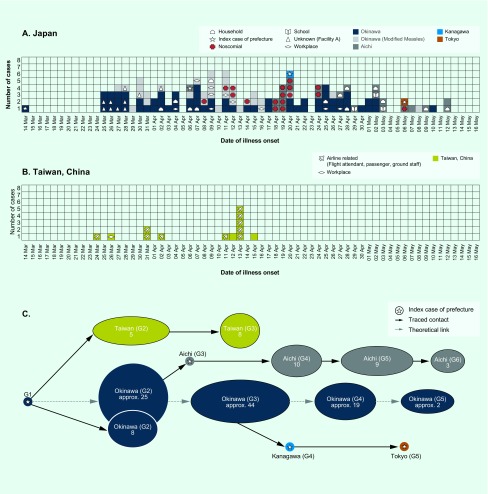
Transmission dynamics of measles in Japan and China, Taiwan, China March–May 2018

**Fig. 2:**
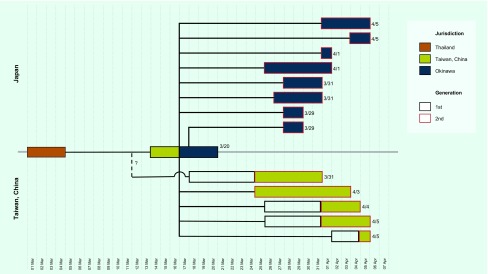
Transmission tree of measles associated with the index case in Japan and China, Taiwan, China, March–May 2018

The cross-border outbreak has been linked to an adult man from China, Taiwan, China who visited Thailand from 1 to 4 March 2018. On 14 March, the index case developed fever and cough. On 17 March, he flew from China, Taiwan, China to Okinawa on a commercial flight, infecting two flight attendants and two passengers who were unvaccinated, and then travelled to Naha city, the capital of Okinawa. The index case developed a rash on 19 March, leading him to seek care at the local medical service. He was hospitalized on the date of clinical diagnosis on 19 March. On 26 March, he returned to China, Taiwan, China on a commercial flight. **Fig. 1** shows the epidemic curves for Japan and China, Taiwan, China. In Japan, at least eight cases were definitively linked to the index case (**Fig. 2**). Many unlinked cases also developed symptoms within a time period consistent with the transmission chain from the index case. In total, the transmission from the index case resulted in producing 123 local cases in Japan and 13 local cases in China, Taiwan, China.

A. Characteristics of measles transmission in Japan given an imported case, March–May 2018. Daily onset of reported measles cases were characterized over time after the onset of illness of the index case on 14 March. Those colored in black (and grey), brown, green and yellow represents measles cases notified respectively in Okinawa, Aichi, Kanagawa, and Tokyo, Japan. Cases from the Okinawa colored in grey were modified measles. Five cases were not accompanied by explicit date of illness onset; dates of illness onset were assumed as the date of confirmation minus five days.

B. Characteristics of an imported measles case in China, Taiwan, China, March–May, 2018. The symptoms of the initial case appeared after arriving to Okinawa, and therefore, this case is not described in B. The squares coloured in green represents cases notified in China, Taiwan, China.

C. Transmission network of the Japan and China, Taiwan, China outbreak of measles, March–May, 2018. The brown box shows the travel period of index case in Thailand, while the following box on the same line represents the illness onset and confirmation of index case. The star indicates the initial case of measles identified in each prefecture of Japan. Numbers in the ellipse represent the number of cases. The bold arrows represent a known link. The dotted arrows represent a theoretical link, assuming that the mean serial interval is 11 days. Note that the horizontal time axes are aligned through panels A, B and C.

Transmission tree of measles in Japan and China, Taiwan, China associated with the index case. Vertical lines represent calendar day. Each box corresponds to a diagnosed case, starting from the date of illness onset to notification. Partially unfilled boxes for four cases in China, Taiwan, China represent an uncertainty of the date of illness onset (i.e. possible range of the date of illness onset). Thick lines represent the link of transmission based on contact-tracing practice. At least eight cases in Japan were linked to the index case.

Ten case patients in China, Taiwan, China were exposed to measles on an aircraft or at airport-associated facilities. One case in China, Taiwan, China acquired an infection in the workplace, and the history of transmission among the other two cases remained unknown. In Japan, 10 cases were infected at an unknown (undisclosed) Facility A in Okinawa prefecture, 17 cases were nosocomial, 13 cases acquired infection in households, 10 cases at their workplace and three were infected in schools. Out of the total 124 cases that include the index case, the route of transmission was unknown for 71 cases.

Due to a large number of transmission events that were very closely traced, the index case is considered to have acted as the primary case of the super-spreading event. The index case visited a densely populated area in Japan (Kokusai Street in Naha city of Okinawa prefecture). In Aichi prefecture, Japan, Nagoya Daini Red Cross Hospital was an important foci of secondary transmissions. In that hospital, a patient who had returned from a trip to Okinawa sought medical attention and unintentionally exposed hospital staff who possessed only low levels of antibodies.

A total of 33 cases in Okinawa prefecture were diagnosed as having modified measles. The transmission potential of modified measles in the current outbreak was estimated elsewhere. (*14*)

### Temporal dynamics in Japan

The first peak of illness was observed on 30 March, and a subsequent peak occurred on 9 April (**Fig. 1A**). Referring to the contact-tracing results of known links and observing the greater variance of illness onset dates in tertiary cases as compared to secondary cases, we determined that there were 30 cases in the fourth generation. Despite intensive follow-up of contacts, 12 cases in the fifth generation and three cases in the sixth generation were observed.

During the third generation, one case patient moved to Aichi prefecture, contributing to 10 subsequent cases in the following generation. **Table 1** shows the composition of cases. The vaccination coverage of case patients aged 9 years old or younger was 33.3%, indicating that they were predominately unvaccinated individuals. Cases in this outbreak were primarily young adults aged 20–39 years (*n* = 70 (56.5%)) (**Table 1**). Cases who had received at least one dose of measles vaccine accounted for 33.1%.

**Table 1:** Age, sex and vaccination history of measles cases in Japan and China, Taiwan, China, March–May, 2018

		Japan Number (%)	Ever vaccinated*	China, Taiwan, China Number (%)
Age (years)	0–9	21 (16.9)	33.3%	-
	10–19	16 (12.9)	43.8%	
	20–29	30 (24.2)	36.7%	7 (53.8)
	30–39	40 (32.3)	37.5%	5 (38.5)
	40–49	13 (10.5)	7.7%	1 (7.7)
	50 and older	4 (3.2)	0.0%	-
Sex	Female	58 (46.8)	37.9%	6 (46.2)
	Male	66 (53.2)	33.3%	7 (53.8)
Measles vaccine received	1+ doses	41 (33.1)	-	-
	0 doses	23 (18.5)	-	-
	Unknown	60 (48.4)	-	13 (100)

* Ever vaccinated indicates the percentage of individuals who have received at least one dose of measles vaccine.

### Temporal dynamics in China, Taiwan, China

**Fig. 1B** shows the epidemic curve in China, Taiwan, China. The index case from the outbreak in Japan also started chains of transmission in China, Taiwan, China. Before travelling to Okinawa, the index case caused a secondary case in a workplace contact. Subsequently, during his flight from China, Taiwan, China to Japan, two flight attendants and two other passengers were infected (**Fig. 1C**). A secondary transmission event was seen on the index case’s flight to Japan, while no one was identified as infected on his way back to China, Taiwan, China (**Fig. 2**). Subsequently, linked to the third and fourth cases, a total of eight tertiary cases were confirmed.

### Estimates of reproduction number

For the first generation, the *R*_n_ in Japan was estimated to be 33.0 (95% CI: 23.0–45.6). In the second generation, the estimate dropped to 1.3 (95% CI: 1.0–1.7); subsequently, the *R*_n_ of the third, fourth and fifth generations took the value below unity, estimated at 0.7 (95% CI: 0.5–1.0), 0.4 (95% CI: 0.2–0.6) and 0.2 (95% CI: 0.0–0.6), respectively. Even when we varied the cut-off date by ± 1 day, the *R*_n_ of the first generation was as large as 37.0 and 28.0, respectively. In China, Taiwan, China, the reproduction number of the first generation was estimated to be 5.0 (95% CI: 1.8–10.7). In the second generation, the *R*_n_ declined to 1.6 (95% CI: 0.7–3.0). Subsequently, cases ceased in China, Taiwan, China, and thus, the *R*_n_ of the third generation was zero.

## Discussion

The present study explored epidemiological features of the cross-border outbreak of measles that spread to Japan and China, Taiwan, China in the WHO Western Pacific Region, where great progress has been made towards measles elimination in recent years. (*3*) As of July 2018, Japan was among eight countries and areas in the Western Pacific Region that had achieved elimination of measles (in addition to Australia, Brunei Darussalam, Cambodia, Hong Kong Special Administrative Region SAR [China], Macao SAR [China], New Zealand and the Republic of Korea). A single index case contributed to super-spreading events, leading us to observe clusters of cases in both Japan and China, Taiwan, China. Given the large number of secondary cases, the chance of observing third and subsequent generations of cases was high even in these highly vaccinated populations. In Japan, the Rn was < 1 only from the third generation, leading the incidence to wane over time. Due to the large outbreak size, a substantial number of contacts were followed, resulting in a resource-demanding outbreak.

Two major conclusions can be drawn from our investigation. First, the outbreak was traced back to a single index case. Potential contributing factors to high individual infectivity include a biological cause, such as an individual who exhales a substantial amount of viruses. In this outbreak, we did not identify a particular risk factor other than contact with the index patient, and the transmission was not restricted to health-care settings. The clinical diagnosis of the index case was swiftly made on the same day of the onset of rash, and many contact events that took place before the onset of rash contributed to secondary transmissions.

Second, given the large number of secondary cases, the clusters of cases did not end up with only one generation of cases even in these highly vaccinated populations. A substantial number of tertiary transmission events were observed. It must be noted that the *R*_n_ of the second generation was estimated to be > 1 for both Japan and China, Taiwan, China. Especially in Japan, the *R*_n_ was < 1 only from the third generation. This event led public health officials to trace a substantial number of contacts in both Japan and China, Taiwan, China, including more than 3500 contacts in China, Taiwan, China alone.

Considering the potential for continued chains of transmission from secondary cases, our study endorses the need to implement supplementary immunization programmes. (*5*, *9*) Three categories of individuals could be susceptible to measles infection and should be targeted for immunization during supplemental campaigns: (1) unvaccinated individuals, especially children; (2) individuals who have received only one dose of measles-containing vaccine; and (3) individuals whose vaccination history is unknown.

The study had several limitations. First, the outbreak in Japan involved a substantial number of modified measles cases whose illness did not meet the definition of a measles case. There could potentially be undiagnosed, modified cases. Second, we used epidemic curves to separate cases into different generations. While we referred to the contact-tracing results, more precise estimation using a sophisticated mathematical modeling approach has yet to be conducted. Third, our study relied on published reports based on the outbreak investigation; more detailed descriptions and discussions over these outbreaks, such as phylogenetic analysis to validate contact-tracing results and detailed laboratory-testing results (e.g. viral load of cases), have yet to be reported.

This study describes an outbreak of measles that originated from a single index case and estimates the *R*_n_. A super-spreading event occurred even with a swift diagnosis upon rash onset. Considering the difficulty with control in this outbreak, our study endorses the importance of vaccinating international travellers, not only those visiting endemic countries but any travellers visiting geographic areas at risk of transmission. To avoid unnecessary chains of transmission, our findings also indicate the importance of continuing and strengthening routine immunization.
